# Real-time visualization of heterotrimeric G protein Gq activation in living cells

**DOI:** 10.1186/1741-7007-9-32

**Published:** 2011-05-27

**Authors:** Merel JW Adjobo-Hermans, Joachim Goedhart, Laura van Weeren, Saskia Nijmeijer, Erik MM Manders, Stefan Offermanns, Theodorus WJ Gadella

**Affiliations:** 1 Swammerdam Institute for Life Sciences, Section of Molecular Cytology, van Leeuwenhoek Centre for Advanced Microscopy, University of Amsterdam, Science Park 904, 1098 XH, Amsterdam, The Netherlands; 2 Nijmegen Centre for Molecular Life Sciences, Department of Biochemistry, Radboud University Nijmegen Medical Centre, Geert Grooteplein 28, 6525 GA Nijmegen, The Netherlands; 3 Department of Chemistry and Pharmaceutical Sciences, Division of Medicinal Chemistry, Leiden/Amsterdam Center for Drug Research, VU University Amsterdam, De Boelelaan 1083, 1081 HV Amsterdam, The Netherlands; 4 Department of Pharmacology, Max-Planck-Institute for Heart and Lung Research, Ludwigstrasse 43, 61231 Bad Nauheim, Germany

## Abstract

**Background:**

Gq is a heterotrimeric G protein that plays an important role in numerous physiological processes. To delineate the molecular mechanisms and kinetics of signalling through this protein, its activation should be measurable in single living cells. Recently, fluorescence resonance energy transfer (FRET) sensors have been developed for this purpose.

**Results:**

In this paper, we describe the development of an improved FRET-based Gq activity sensor that consists of a yellow fluorescent protein (YFP)-tagged Gγ2 subunit and a Gαq subunit with an inserted monomeric Turquoise (mTurquoise), the best cyan fluorescent protein variant currently available. This sensor enabled us to determine, for the first time, the k_on _(2/s) of Gq activation. In addition, we found that the guanine nucleotide exchange factor p63RhoGEF has a profound effect on the number of Gq proteins that become active upon stimulation of endogenous histamine H1 receptors. The sensor was also used to measure ligand-independent activation of the histamine H1 receptor (H1R) upon addition of a hypotonic stimulus.

**Conclusions:**

Our observations reveal that the application of a truncated mTurquoise as donor and a YFP-tagged Gγ2 as acceptor in FRET-based Gq activity sensors substantially improves their dynamic range. This optimization enables the real-time single cell quantification of Gq signalling dynamics, the influence of accessory proteins and allows future drug screening applications by virtue of its sensitivity.

## Background

Heterotrimeric G proteins are composed of Gα subunits and Gβγ dimers, and can be activated by G-protein-coupled receptors (GPCRs). Upon binding of an agonist, the receptor changes its conformation, and acts as a guanine nucleotide exchange factor (GEF). By inducing the exchange of guanine diphosphate (GDP) for guanine triphosphate (GTP) in the Gα subunit, the G protein becomes active [1]. Gβγ interacts with Gα through two interfaces: the switch region and the region formed by the N terminus of the Gα subunit. Binding of GTP upon receptor activation disrupts the switch interface, which is thought to trigger the dissociation of Gβγ from Gα [2]. The family of Gα subunits consists of four classes; the subunits Gαq, Gα11, Gα14 and Gα16 belong to the Gq class. The principal target of the Gq class is phospholipase (PL)Cβ [3]. Recently, RhoGEF proteins such as leukemia-associated Rho-guanine nucleotide exchange factor (LARG) and p63RhoGEF have been shown to directly interact with and to be regulated by Gαq, suggesting that they can link Gαq-coupled receptors to the activation of the small G protein RhoA [4-6].

Studying Gαq is important, because this protein is implicated in the development of myocardial hypertrophy after mechanical stress of the heart [7,8]. Because this is one of the triggers of cardiac failure, a leading cause of death in the western world, drugs to inhibit Gαq are much in demand [9]. Proteins belonging to the Gq class are also involved in the modulation of synaptic transmission [10,11], cell growth, platelet aggregation [12], glucose secretion, actin cytoskeletal rearrangements, hematopoietic cell differentiation, leukocyte activation and contraction of smooth muscle, emphasizing their importance in human physiology [13].

Recently, several fluorescence resonance energy transfer (FRET) sensors have been developed to monitor the activation state of specific heterotrimeric G proteins in living cells upon GPCR activation [14-21]. In this paper, we report on the development of a highly sensitive sensor based on functional mTurquoise-tagged Gαq and yellow fluorescent protein (YFP)-tagged Gγ2, which allows for monitoring of the location and G protein activation state of Gq in living cells and of the kinetics of the process. In addition, we describe the effects of ligand-dependent and ligand-independent stimulation of endogenous and overexpressed receptors, while concurrently monitoring the influence of effectors on the behaviour of the sensor. We opted for dual emission ratiometric FRET measurements supplemented with FRET-fluorescence lifetime imaging microscopy (FLIM) measurements to monitor the kinetics of Gq activation and FRET efficiencies, respectively.

## Results

### Construction of a Gq FRET sensor

**Figure 1:**
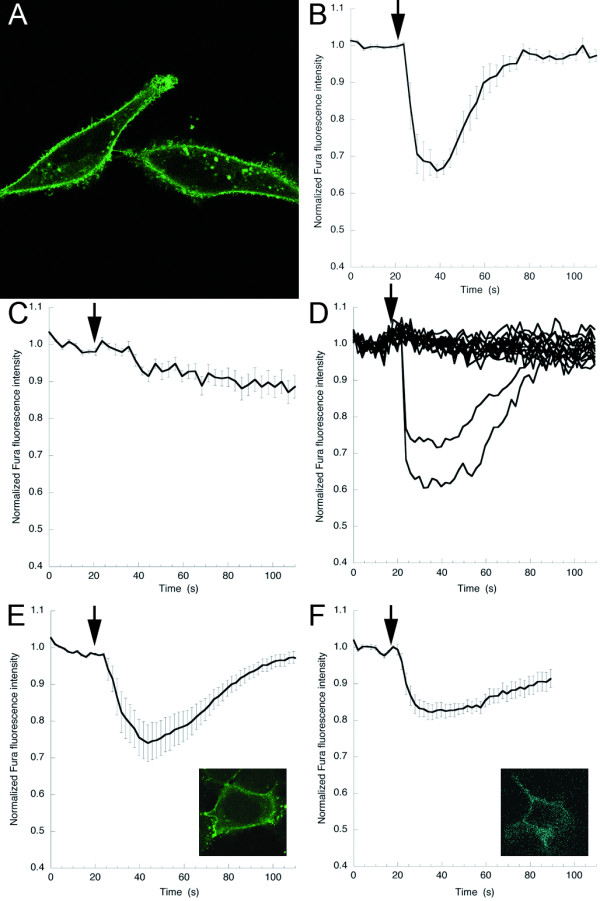
**Gαq-visible fluorescent protein (VFP) is functional**. **(A) **HeLa cells expressing Gαq tagged with monomeric yellow fluorescent protein (Gαq-mYFP). **(B) **Mouse embryonic fibroblasts (MEFs) derived from wild-type mice showed an increase in [Ca^2+^]_i _upon addition (arrow) of bradykinin (BK) (1 μmol/l, n = 6. Error bars depict SE). **(C) **MEFs derived from Gαq/11-deficient mice (MEFq/11^-/-^) did not display increased [Ca^2+^]_i _upon addition of BK (n = 9). **(D) **Expression of wild-type Gαq in MEFq/11^-/- ^caused an increase in cytosolic calcium upon addition of BK (n = 2). Most cells did not express the re-introduced wild-type (untagged) Gαq and did not show a decrease in Fura Red intensity upon addition of BK. **(E) **Expression of Gαq-mYFP in MEFq/11^-/- ^caused an increase in cytosolic calcium upon addition of BK (n = 12); (Inset) MEFq/11^-/- ^cell expressing Gαq-mYFP. **(F) **Gαq-mTqΔ6 similarly caused an increase in [Ca^2+^]_i _upon addition of BK (n = 6). (Inset) MEFq/11^-/- ^cell expressing Gαq-mTqΔ6.

To further our understanding of the kinetics of Gq signalling in living cells, we prepared visible fluorescent protein (VFP)-tagged human Gαq subunits. Because neither N- nor C-terminal fusions of Gαs to VFP retained functionality [22,23], we opted for insertion of the fluorescent protein. The VFP insertion site was based on a Gαq tagged with hemagglutinin (HA) in the α-helical domain (residues 125-ENPYVD-130 replaced by DVPDYA [24,25]) and on green fluorescent protein tagged Gαq (Gαq-GFP), as described previously [26]. Monomeric (by applying the A206K mutation [27]) YFP (mYFP) was inserted into the αB-αC loop of the Gαq subunit (see Methods section for more details). Upon transient expression, the protein was found at the plasma membrane, as judged from the fluorescence pattern seen in HeLa cells (Figure 1A). Fluorescence was also, to variable extents, detected in the cytoplasm and occasionally in the nucleus. In various other cell lines (Madin-Darby canine kidney (MDCK), N1E-115 neuroblastoma and HEK293) a similar pattern was seen (see Additional file 1A-C). The localization pattern of Gαq-GFP has been thoroughly described by Berlot and colleagues [26], and is comparable with the Gαq-mYFP variant reported here.

FRET, which is a distance- and conformation-dependent phenomenon, is very useful for the study of dynamic protein interactions in living cells [28]. To measure FRET, various techniques can be used. To investigate the kinetics of the interaction between Gαq and the Gβγ dimer, FRET ratio imaging with a cyan fluorescent protein (CFP)-YFP pair was the method chosen. Ratiometric imaging is an intensity-based technique to analyze FRET, which involves measurement of the ratio between donor and acceptor fluorescence intensity after induction of signal transduction [29]. Recently, we reported a novel bright CFP variant, monomeric Turquoise (mTq), which has excellent properties for use in FRET imaging. This fluorescent protein is a monomeric CFP variant that is twice as bright as enhanced (E)CFP, is more photostable, displays an improved signal:noise ratio for ratiometric FRET measurements, has a long fluorescent lifetime, and decays mono-exponentially [30]. Therefore, we replaced the mYFP with mTq in the Gαq-mYFP construct to use it as a donor in FRET studies.

Since mTq is inserted and the N and C termini of mTq are on the same side of the β-barrel, we reasoned that a C-terminal deletion of six amino acids (mTqΔ6, yielding a fluorescent mTq as in yellow cameleon [31]) would effectively bend the mTq moiety in Gαq, thereby forcing the mTq fluorophore (and its dipole moment) in another orientation. This could have a profound effect on the FRET efficiency (via the κ^2 ^orientation factor, [28]). Importantly, this variant was also found at the plasma membrane in HeLa cells, similar to Gαq-mYFP (see Additional file 1D).

We chose to tag the N terminus of Gγ2 with YFP, to effectively label the Gβγ dimer with an acceptor, as this fusion has previously been shown to retain functionality [32].

### Functionality of the Gq sensor

The functionality of the fluorescent Gαq proteins (Gαq-mYFP and Gαq-mTqΔ6) was tested in a mouse embryonic fibroblast (MEF) cell line prepared from Gαq/11-deficient mice [33]. This cell line (MEFq/11^-/-^) and a MEF cell line prepared from wild-type mice express the bradykinin (BK) type 2 receptor that couples to Gαq [34]. As expected, addition of BK (1 μmol/l) led to an increase in cytosolic calcium in the wild-type MEF cell line (Figure 1B), but not in the MEFq/11^-/- ^cell line (Figure 1C). Expression of wild-type Gαq (Figure 1D), Gαq-mYFP (Figure 1E) or Gαq-mTqΔ6 (Figure 1F) in MEFq/11^-/- ^cells caused an increase in cytosolic calcium upon addition of BK. These results indicate that Gαq-mYFP and Gαq- mTqΔ6 can substitute for the wild-type Gαq in living cells devoid of endogenous Gαq/11 proteins. Using western blot analysis (see Additional file 2), we compared the expression level of endogenous Gαq/11 in MEF cells from wild-type mice with that of Gαq-mYFP transduced into MEFq/11^-/-^. Gαq-mYFP in MEFq/11^-/- ^was clearly less abundant than Gαq/11 in wild-type MEF cells, indicating that the observed calcium responses are not simply due to overexpression.

Correct localization of the heterotrimer requires cotransfection of the three subunits, as described previously [35]. To examine whether Gαq-mTq forms a heterotrimer with the YFP-tagged Gβγ dimer, steady-state FRET efficiencies were measured in cells coexpressing Gαq-mTq and YFP-Gγ2. The truncated version, Gαq-mTqΔ6, displayed a higher basal FRET level (FRET efficiency 26%; see also Figure 6) in the presence of YFP-Gγ2, compared with the non-truncated form (FRET efficiency 18%; data not shown) and was therefore used in Gq activation measurements.

### Dynamic range of the Gq sensor

During the course of our studies, Jensen *et al. *[19] published data on Gq activation using FRET ratio imaging with Gαq-ECFP and EYFP-Gβ1. We compared the expression of Gαq-ECFP with that of Gαq-mTqΔ6 by transfecting cells with similar quantities of DNA. Cells expressing Gαq-mTqΔ6 were readily visible, whereas cells expressing Gαq-ECFP were substantially less fluorescent (see Additional file 3).

**Figure 2:**
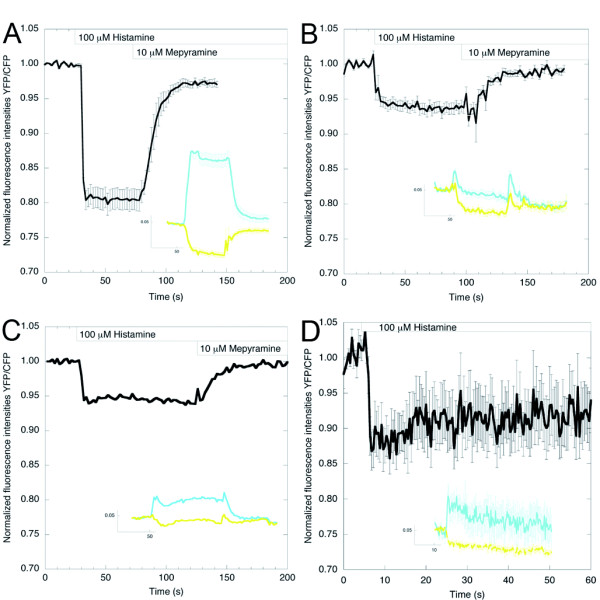
**Dynamic range of Gq sensors**. **(A) **The fluorescence resonance energy transfer (FRET) ratio change (yellow fluorescent protein:monomeric Turquoise (YFP:mTq)) upon addition of histamine (100 μmol/l) in HeLa cells coexpressing Gαq-mTqΔ6, Gβ1, YFP-Gγ2 and the histamine H1 receptor (H1R) (Inset) the mTq and YFP intensity traces from which the ratio was derived (n = 14; error bars depict SE). Addition of the H1R inverse agonist mepyramine (10 μmol/l) reversed the ratio change induced by histamine. **(B) **The FRET ratio change (YFP:CFP) upon addition of histamine (100 μmol/l) in HeLa cells coexpressing Gαq-enhanced (E)CFP, EYFP-Gβ1, Gγ2 and H1R. (Inset) the CFP and YFP intensity traces from which the YFP:CFP ratio was derived (n = 17; error bars depict SE). **(C) **Representative trace of the FRET ratio change (YFP:mTq) upon addition of histamine (100 μmol/l) in HeLa cells coexpressing Gαq- mTqΔ6, EYFP-Gβ1, Gγ2 and H1R. (Inset). The mTq and YFP intensity traces from which the ratio was derived. **(D) **The FRET ratio change (YFP:CFP) upon addition of histamine (100 μmol/l) in HeLa cells coexpressing Gαq-ECFP, Gβ1, YFP-Gγ2 and H1R. (Inset) the CFP and YFP intensity traces from which the ratio was derived (n = 5; error bars depict SE).

To determine the dynamic range of our Gq activity sensor, we overexpressed the histamine H1 receptor (H1R) in HeLa cells, and compared the amplitude of the ratio change of our and the previously described sensor [19] under identical conditions (Figure 2A,B). We examined the expression of H1R and Gαq-mTqΔ6 under these conditions. Binding studies with a radiolabeled ligand revealed 710 fmol/mg binding sites, which is about fivefold higher than that of wild-type levels (150 fmol/mg for mock-transfected HeLa cells). Western blotting (see Additional file 2) indicated that Gαq-mTqΔ6 was expressed at a level equal to the endogenous Gαq protein.

Addition of histamine caused intensity changes with much larger amplitudes (about twice as high) in our Gq sensor. The intensity traces unequivocally showed that, upon stimulation, the intensity of the donor (mTq) increased, whereas the sensitized emission of the acceptor (YFP) decreased (Figure 2A, inset), indicating a loss of FRET and reflecting an increase in distance between the αB-αC loop of Gαq and the N terminus of Gγ2, or a change in orientation. These clear, reciprocal changes in CFP and YFP intensities were not seen for the other sensor under identical circumstances (Figure 2B, inset). The results confirm the improved fluorescence properties of mTurquoise [30] and the benefits of deleting the six C-terminal amino acids of this tag.

Addition of the H1R inverse agonist mepyramine [36-38] readily caused deactivation (Figure 2; see Additional file 4). Under H1R overexpression conditions and in the continued presence of histamine in the medium, activation of Gq remained unaltered for at least 8 minutes (see Additional file 5). Similar changes were seen upon overexpression of the BK receptor type 2 (data not shown).

To ascertain the effect of using YFP-Gγ2 as acceptor instead of EYFP-Gβ1, we determined the FRET change in cells coexpressing Gαq-mTqΔ6 and EYFP-Gβ1 (Figure 2C). Using EYFP-Gβ1 as acceptor clearly diminished the amplitude of the response, indicating that the use of YFP-Gγ2 as an acceptor increases the sensitivity of the sensor. When Gαq-ECFP was used together with the acceptor YFP-Gγ2, a more robust FRET change was seen (compare Figure 2D with 2B). In summary, these data show that the combination of Gαq-mTqΔ6 and YFP-Gγ2 significantly improve the response of the Gq activity sensor.

**Figure 3:**
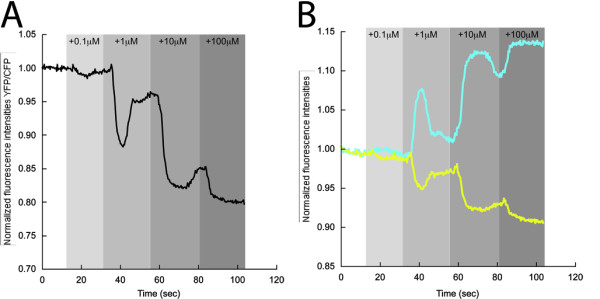
**Dose-response curve**. **(A) **Representative trace depicting the fluorescence resonance energy transfer (FRET) ratio changes (yellow fluorescent protein:monomeric Turquoise (YFP:mTq)) upon addition of increasing amounts of histamine (gray bars) in HeLa cells expressing Gαq-mTqΔ6, Gβ1, YFP-Gγ2 and the histamine H1 receptor (H1R). **(B) **The mTq and YFP intensity traces from which the ratio was derived.

To investigate the sensitivity of our sensor (Gαq-mTqΔ6/YFP-Gγ2), we overexpressed H1R and tested the response of the sensor to a range of agonist concentrations (Figure 3). Interestingly, a transient FRET change was seen upon stimulation with 0.1 μmol/l histamine, whereas its duration was prolonged upon addition of higher amounts of histamine. It seems that the amount of active Gq accumulates progressively every time the total concentration of histamine is increased, and that concomitantly, active Gq is desensitized less efficiently at each new histamine addition.

### Kinetics of Gq activation

**Figure 4:**
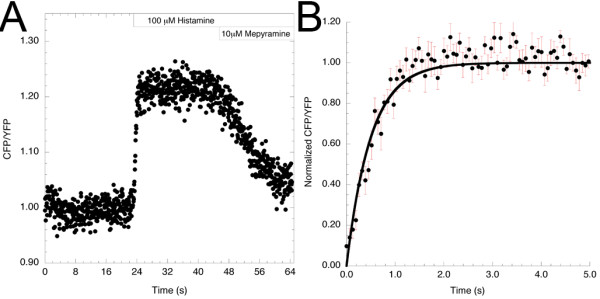
**Kinetics of Gq activation**. **(A) **The fluorescence resonance energy transfer (FRET) ratio change (yellow fluorescent protein:monomeric Turquoise (YFP:mTq)) upon addition of histamine in cells expressing Gαq-mTqΔ6, Gβ1, YFP-Gγ2 and histamine H1 receptor (H1R), measured with high temporal resolution. Images were acquired at a frame rate of 0.064 seconds, and 1000 data points are shown. **(B) **Average FRET ratio change (scaled between 0 and 1) upon addition of histamine (n = 11; error bars depict SE). The data points were appropriately fitted with a monoexponential curve (solid line).

An important parameter describing the Gq activation kinetics is its activation rate (k_on_) upon receptor activation. To date, no conclusive data has been produced to quantify this kinetic parameter. By monitoring the FRET change kinetics of the FRET pair (Gαq- mTqΔ6 and YFP-Gγ2) upon addition of histamine, we were able to determine the activation kinetics of the heterotrimer. Activation of Gq was too fast to be measured by our widefield set-up (which used sequential CFP/YFP acquisition), therefore the experiments were repeated using a laser-scanning microscope with a resonant scanner. With this, it was possible to perform ratio imaging of FRET with a frame rate of around 15 frames/s, which provided sufficient temporal resolution to measure the kinetics of Gαq activation by H1 receptors (Figure 4A). The activation kinetics was appropriately fitted using a monoexponential curve, yielding a half-time for activation of 350 ms (Figure 4B), implying that k_on _= 2/s.

### Influence of accessory proteins on the activation state of Gq

**Figure 5:**
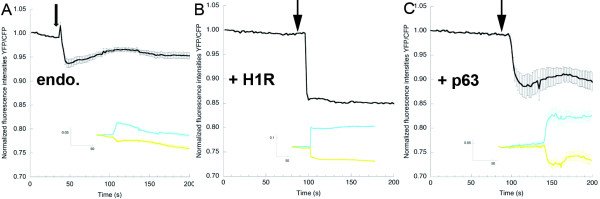
**Accessory proteins affect the activation state of Gq**. **(A) **endo.: the fluorescence resonance energy transfer (FRET) ratio change (yellow fluorescent protein:monomeric Turquoise (YFP:mTq)) upon addition (arrow) of histamine (100 μmol/l) in HeLa cells expressing Gαq-mTqΔ6, Gβ1 and YFP-Gγ2. (Inset) the mTq and YFP intensity traces that belong to the ratio (n = 9; error bars depict SE). **(B) **+H1R: the FRET ratio change (YFP/mTq) upon addition (arrow) of histamine (100 μmol/l) in HeLa cells expressing Gαq-mTqΔ6, Gβ1, YFP-Gγ2 and histamine H1 receptor (H1R). (Inset) the mTq and YFP intensity traces that belong to the ratio. **(C) **+p63: the FRET ratio change (YFP/mTq) upon addition of histamine (100 μmol/l) in HeLa cells expressing Gαq-mTqΔ6, Gβ1, YFP-Gγ2 and the guanine nucleotide exchange factor p63-RhoGEF. (Inset) the mTq and YFP intensity traces that belong to the ratio (n = 9).

Besides studying activation kinetics under receptor overexpression conditions, we were interested in obtaining data in the absence of overexpressed receptors. Stimulation of the endogenous [39] histamine receptor type 1 (H1R) in HeLa cells by addition of histamine also led to a measurable FRET decrease (Figure 5A), but with markedly different kinetics. Because Gα subunits cycle between their inactive (GDP-bound) and active (GTP-bound) states, the duration of the active state is limited by its intrinsic GTP hydrolysis and GTP-ase activating protein (GAP) activity executed by accessory proteins. Remarkably, even in the continuous presence of agonist, the activation state of Gq is partially desensitized (Figure 5A), contrary to reports for Gαi [16] and Gαs (albeit in the presence of overexpressed receptors) [17]. Clearly, under H1R overexpression conditions (Figure 2A; Figure 5B; see Additional file 5), the activation state of Gq is prolonged. Gαq can be stimulated to hydrolyze its bound GTP by both regulators of G-protein signalling (RGS) proteins and phospholipase (PL)Cβ isozymes [40,41]. To test the role of RGS proteins in desensitization of active Gq, an RGS-insensitive Gαq-mTqΔ6 was constructed by mutating G188 in switch I into S188. This mutation has been shown to inhibit binding of RGS proteins to Gαq ([42] and references therein), while leaving the intrinsic rate of GTP hydrolysis unchanged. FRET measurements using Gαq-mTqΔ6-G188S and YFP-Gγ2 show that the onset of Gq activation is slower. Desensitization also seems to be compromised, albeit not completely absent (see Additional file 6).

Next, we studied the influence of the recently described effector of Gq, p63RhoGEF [5,6,43], on the activation state of Gq. Strikingly, coexpression of p63RhoGEF again caused an increase in the observed ratio changes between Gαq-mTqΔ6 and YFP-Gγ2 upon addition of histamine (Figure 5C). The decrease in FRET readily returned to baseline upon addition of mepyramine (see Additional file 7). Importantly, the Gαq-interaction mutant p63RhoGEF-L475A [43] did not induce such an increase (see Additional file 8).

The prolonged FRET response with stimulation in the presence of p63RhoGEF or overexpressed H1R enabled us to apply the more quantitative FLIM technique to measure FRET levels. With FLIM, the excited state lifetime of the donor fluorophores is determined. In the case of FRET, the excited state duration is shortened because of transfer of energy to the acceptor [44]. Because fluorescence lifetimes do not depend on local excitation intensity or fluorophore concentration, and are largely unaffected by moderate levels of photobleaching of the fluorophores, FLIM is a very robust method to quantify FRET in living cells [29]. In the case of frequency-domain FLIM, two lifetimes can be measured, that is, the phase lifetime and the modulation lifetime, which both decrease in FRET.

**Figure 6:**
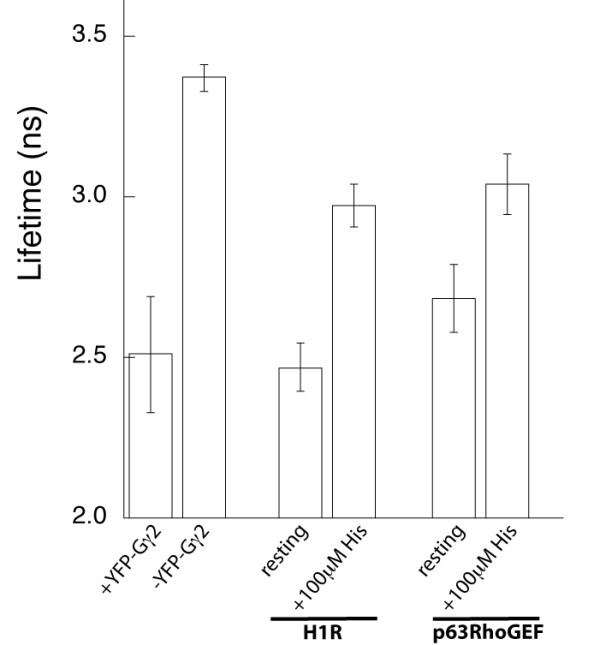
**Fluorescence lifetime data**. Average cyan fluorescent protein (CFP) lifetimes measured under different conditions. The phase lifetime of Gαq-mTqΔ6 was measured in the absence (n = 15) and presence (n = 12) of the acceptor YFP-Gγ2. The difference in lifetime was significant. The Gq sensor was coexpressed with the histamine H1 receptor (H1R) and CFP lifetimes were measured before and after addition of 100 μmol/l histamine (n = 6). The Gq sensor was coexpressed with the guanine nucleotide exchange factor p63RhoGEF and CFP lifetimes were measured before and after addition of 100 μmol/l histamine (n = 6). In both conditions the lifetime difference before and after stimulation was significant. The CFP lifetimes before addition of histamine are not different (Student *t*-test, 95% CI) in the presence or absence of either H1R or p63RhoGEF.

The FLIM data revealed a reduction of the mTurquoise modulation lifetime (data not shown) and phase lifetime from 3.4 ns (donor only) to 2.5 ns in the presence of its acceptor YFP-Gγ2 (Figure 6). These lifetimes correspond to a FRET efficiency of 26%. In the presence of H1R, similar FRET efficiencies were seen in resting cells, but upon addition of histamine, the donor lifetime increased. Similar increases were found by stimulating cells coexpressing p63RhoGEF with the Gq FRET sensor. These results unequivocally showed a decrease in FRET in the Gq sensor upon GPCR stimulation (Figure 6; see Additional file 9), corroborating the ratiometric data.

### Ligand-independent activation of H1R activates Gq

**Figure 7:**
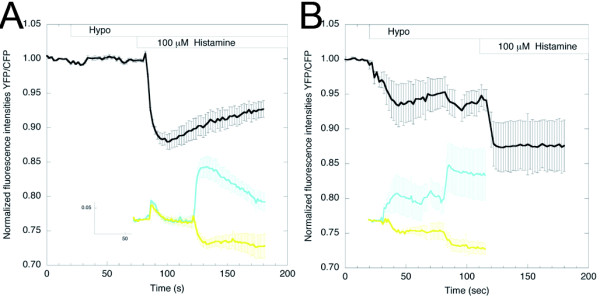
**Ligand-independent activation of Gq**. **(A) **Graph depicting the fluorescence resonance energy transfer (FRET) ratio (yellow fluorescent protein:monomeric Turquoise (YFP:mTq)) change upon the application of a hypotonic (Hypo) stimulus, followed by the addition of histamine (100 μmol/l) in HeLa cells expressing Gαq-mTqΔ6, Gβ1 and YFP-Gγ2 (n = 5; error bars depict SE). **(B) **HeLa cells overexpressing the H1 receptor showed significant activation of Gq when the osmolality of the medium was reduced (Hypo). Subsequent addition of histamine yielded additional activation of Gq (n = 5).

Recently, Mederos y Schnitzler *et al. *[45] reported that certain GPCRs function as sensors of membrane stretch, with the H1 receptor being particularly mechanosensitive compared with AT1R, M5R and V1AR. Membrane stretch can be induced by the application of a hypotonic stimulus (hypotonic cell swelling). First, we examined whether hypotonic stimulation of endogenous H1 receptors of HeLa cells could induce Gq activation, and found that it was not sufficient to produce a measurable Gq activation, as a ratio change was not seen (Figure 7A). Importantly, the same cells were capable of responding to histamine (Figure 7A).

Next, we examined whether overexpression of H1 receptors could trigger Gq activation upon application of a hypotonic stimulus. A clear FRET decrease was seen in these cells (Figure 7B), indicating Gq activation. These results suggest ligand-independent activation of mechanosensitive H1R, leading to the activation of Gq. The activation of Gq, as detected by the decrease in FRET, led to activation of PLCβ. We visualized the activity of PLC by monitoring the PH domain of PLCδ1 (PHδ1), which specifically binds PtdIns(4,5)P_2 _[46]. Addition of a hypotonic stimulus caused the PHδ1 domain to translocate from the plasma membrane to the cytosol, indicative of the hydrolysis of PtdIns(4,5)P_2 _(see Additional file 10). Consecutive addition of mepyramine did not affect the localization of PHδ1, suggesting that the activation of H1R by stretch cannot be inhibited by an antagonist.

## Discussion

### Characteristics of the Gq FRET sensor

To evaluate functionality, we extensively tested the VFP-tagged Gq in MEF cells deficient in Gq/11. The results in these cells showed that Gαq-mTqΔ6 effectively interacts with GPCRs and PLCβ (Figure 1). In addition, we can state that it can also form heterotrimers with Gβγ subunits, because receptors bound Gα subunits poorly and failed to trigger GDP exchange in the absence of Gβγ [47]. Moreover, we saw substantial FRET when Gαq-mTqΔ6 was coexpressed with an YFP-tagged Gγ2.

The spatiotemporal characteristics of heterotrimeric G protein activation are difficult to monitor, because direct measurement of GTP binding and hydrolysis is not feasible in living cells [48]. Instead, an increase in intracellular calcium, measured using calcium-sensitive fluorescent probes, is often used as an indicator of Gq activity. However, other heterotrimeric G proteins can cause increases in intracellular calcium by means of calcium entry from the extracellular environment, and by activation of PLCβ via Gβγ dimers [49-51]. In addition, increases in intracellular calcium triggered by Gq-coupled receptors are amplified signals that are modulated by downstream factors (for example, calcium-store depletion and regulation of effector function by Ca^2+^-activated kinases). Hence, they are not ideal to quantify the extent and dynamics of G protein activation. The FRET pair described here enables the direct measurement of heterotrimeric G protein activation. Through the use of an optimized fluorophore, the bright CFP variant mTurquoise (see Additional file 3), positioned in an orientation yielding a higher basal FRET efficiency level in the intact heterotrimer, in combination with YFP-Gγ2 as an acceptor, we were able to measure Gq activation kinetics in single cells with high temporal resolution.

The half-time of Gq activation of 350 ms is faster than those of Gs and Gi (450 and 690 ms, respectively) (Figure 4) [14,17]. As receptor-G-protein interaction has been shown to be maximal after about 50 ms for several GPCR-G-protein pairs [15,17,52], it is likely that the H1R-Gq interaction displays similar kinetics. Only a few Gq heterotrimers will be active at this early time point. Full activation of Gq proteins requires several hundred milliseconds, making G-protein activation (not receptor activation, receptor diffusion or receptor-G protein interaction) a limiting step in GPCR activation of downstream effectors. Interestingly, Mukhopadhyay and Ross [53] described the kinetics of Gq activation *in vitro*, and found GTP exchange to be rate-limiting for Gq activation. However, at cellular GTP levels, the GDP dissociation rate became rate-limiting. The GDP dissociation rate was found to be 1.5/s, which correlates well with the rate of Gq activation determined by means of our FRET sensor in living cells.

Previous studies with FRET sensors on activation of other classes of heterotrimeric G proteins showed a sustained activity in the continued presence of agonist [14-17,19,52]. For example, Gi activation only desensitizes noticeably upon removal of the agonist or addition of specific antagonists [14,16]. In our study, we also saw a long-lasting Gq activation in the presence of agonist when the histamine receptor was overexpressed (see Additional file 5), but the activation was readily reversed when an antagonist was added. By contrast, Gq activation was partially desensitized when endogenous histamine receptors were activated (Figure 5A). Presumably, this recovery is possible because GAPs are able to convert Gαq-GTP into Gαq-GDP under these conditions. Indeed, upon introduction of G188S in Gαq-mTqΔ6, the Gq activation kinetics changed compared with those of Gαq-mTqΔ6; the response had a slower onset and prolonged activation (see Additional file 6). Clark and Lambert also described these phenomena previously [42]. However, in our case the observed phenotype was rather weak, which may imply that PLCβ is the physiological GAP in our system. Importantly, the dose-response curve analysis (Figure 3) showed that the FRET change was also transient at low agonist concentrations under conditions in which histamine receptor was overexpressed. The histamine levels we used are in the physiological range, that is, whole blood levels were 25-65 ng/ml of histamine [54], corresponding to histamine concentrations in whole blood of 0.22-0.58 μmol/l. Our data suggest that cells overexpressing H1R may show a small, transient Gq activation response to these histamine levels in blood.

### Influence of accessory proteins on the activation state of Gq

Overexpression of p63RhoGEF led to an increase in the FRET ratio amplitude (Figure 5C), but the Gαq-interaction mutant, p63RhoGEF-L475A, did not have an effect on the amplitude (see Additional file 8). It is likely that p63RhoGEF retains Gq in its active state by shielding it from the GAP PLCβ, thereby accumulating Gαq-GTP.

The question of whether intact heterotrimers or dissociated G-proteins (Gαq and Gβγ) activate downstream effectors has not been resolved to date, and most of our knowledge has been obtained from structural studies with purified proteins. Gαq-GDP and Gβγ interact through two interfaces. The most extensive interface is formed by residues close to and within switch I and II from the Gα subunit. These residues contact Gβ residues at the top of the propeller domain. The second interface is formed by the N terminus of Gα and the side of the Gβ propeller [2,55]. Gβ is most severely affected in binding to Gα when it contains mutations in the region that interacts with the N terminus of Gα, indicating that the N terminus is crucial in mediating the interaction with Gβ [56]. It may therefore be envisioned that, upon activation, the contact of Gβ with the switch regions is lost, whereas its contact with the N terminus remains, as suggested by Gibson and Gilman [16]. This would enable an opening up of the trimer, allowing Gα and Gβγ to interact with their downstream signalling partners. However, activation of certain effectors may require complete dissociation of the heterotrimer. Accessory proteins may cause dissociation of Gαq and Gβγ by forcing them apart. The recently published crystal structure of the G protein-coupled receptor kinase GRK2 in complex with Gαq-GTP and Gβγ exemplifies this idea [57]. It showed that GRK2 interacts with Gβγ residues implicated in both interfaces, thereby probably causing trimer dissociation.

The increases in FRET ratio amplitude that we observed may be due to an increase in fully dissociated heterotrimers, which causes a loss of FRET. However, a loss in FRET can also be explained by a change in orientation between donor and acceptor fluorophore via the k^2 ^factor in the equation that determines the Förster radius. Interestingly, even under conditions of maximal GEF and minimal GAP activity, we detected extensive FRET (20%) between Gαq and Gγ2, indicating that the majority of Gαq is still in close proximity to Gγ2 (on average in the order of 7 nm, based on the FRET Förster radius). These data would support the partial opening hypothesis as outlined above, implying that full dissociation does not occur. However, although it indicated that Gq is not fully dissociated under these maximal stimulation conditions, our method cannot distinguish between a partial dissociation leaving a fraction of non-dissociated heterotrimers with unaltered high FRET efficiency (25%) and a full conformational change of all heterotrimers characterized by a lower FRET efficiency. Importantly, others have proposed a signalling role for the intact Gq heterotrimer [58].

### Ligand-independent activation of H1R activates Gq

Ligand-independent activation of GPCRs is a new and not yet fully explored phenomenon. It was described by Mederos y Schnitzler *et al. *[45] in HEK293 cells and COS-7 cells overexpressing the histamine H1 receptor, among others. Conformational changes in GPCR (FRET-based measurements) upon addition of a hypotonic stimulus and consecutive receptor activation have been described for BK2R in bovine aortic endothelial cells [59], for PTH1R (FRET-based) in pre-osteoblastic cells [60], and for AT1R (determined by means of substituted cysteine accessibility mapping) in HEK293 cells [61]. Studying mechanotransduction is especially important with respect to its effects on the cardiovascular system. For instance, mechanical stress to cardiomyocytes can trigger hypertrophic responses [62]. Using our Gq activity sensor, we were able to monitor Gq activation upon application of a hypotonic stimulus. Overexpression of H1R was required to observe a ratio change. Interestingly, we did observe an increase in the ratio change of the Gq FRET sensor in the presence of endogenous H1R upon addition of histamine, after hypotonic swelling of the cells. This suggests that heterotrimeric G proteins are triggered to bind the endogenous H1 receptor upon hypotonic stress, which may lead to changes in orientation of or distance between a small fraction of trimers. This pre-accumulation at the H1R might explain the larger amplitude seen upon addition of histamine, compared with that seen without prior exposure to hypotonic stress (Figure 5A).

Determination of H1R expression levels showed that in our cells overexpression produced an average of 710 fmol/mg binding sites. Similarly, in A7r5 cells derived from the aorta (a mechano-insensitive tissue) overexpression of the AT1 receptor of about 15 times (88 fmol/mg versus 1305 fmol/mg) induced mechanosensitivity [45]. Moreover, the expression of AT1R in the aorta has been described to be 5 fmol/mg [63], whereas it can be up to 200 fmol/mg in the afferent arterioles (a mechanosensitive tissue) [64].

A conformational change in the AT1 receptor upon mechanical stress has been described [61]. Currently, it is not clear how this conformational change is triggered, and whether all mechanosensitive GPCRs undergo a similar change in conformation. It is likely that a FRET approach may shed more light on this matter.

## Conclusions

By virtue of the optimized Gq FRET sensor described in this paper, the activation of Gq can now directly be measured with high spatial and temporal resolution in single living cells without having to rely on pharmacological inhibitors to delineate the involvement of specific signalling pathways. As a result we have determined k_on _of Gq for the first time. It is likely that FRET-based sensors for the measurement of other classes of G proteins, which employ ECFP as donor and EYFP-Gβ1 as acceptor, will be substantially improved by incorporating (truncated and monomerized) mTurquoise and using YFP-Gγ2 as acceptor.

Inhibitors of G proteins are attractive drug candidates [47]. The fact that overexpression of the C terminus of Gαq has a positive effect on cardiac hypertrophy indicates a possible strategy for preventing pathophysiological signalling [7]. The FRET pair we developed may aid in the search for new drugs by facilitating highly sensitive Gq activity measurements in living cells. Moreover, we have shown that this tool can be used to study the kinetics of heterotrimeric G protein signalling and the influence of accessory proteins.

## Methods

### DNA constructs

The pcDNA3.1+ plasmids containing the cDNA encoding human Gαq, Gβ1, Gγ2 and H1R were obtained from the Missouri S&T cDNA Resource Center (http://www.cdna.org). We inserted monomeric (A206K) spectral variants of GFP in the αB-αC loop of Gαq in between residues 127 and 128, thereby changing N126 into D126. The linkers present in the Gαq-YFP construct are SGGGGS at the N terminus of the FP and SGGSGD at the C terminus of the FP. To make the Gαq-mTqΔ6 construct, the last six amino acids of mTq were removed. The linkers present in this construct are SGGGGS at the N terminus of the FP and I at the C terminus of the FP. Gαq-YFP was amplified by means of PCR and inserted into the retroviral vector pBABE-puromycin (Addgene Inc, Cambridge, MA, USA). Detailed sequence information of the construct is available upon request.

To make YFP-Gγ2, we amplified Gγ2 by means of PCR and cloned it behind circular-permutated Venus derived from YCam3.60 [65] (kind gift of Dr A Miyawaki, Riken, Wako, Japan). The full-length p63RhoGEF was a gift from Dr J Sondek (University of North Carolina, Chapel Hill, NC, USA); Gαq-ECFP was provided by Dr C. Berlot (Weis Center for Research, Danville, PA, USA)and EYFP-Gβ1 by Dr S Ikeda (National Institutes of Health, Bethesda, MD, USA). Gαq-ECFP was transferred from pcDNAI to a high copy number plasmid by replacing EGFP in the pEGFP-C1 plasmid, using the restriction enzymes *Sna*BI and *Psp*OMI.

### Cell culture and transfection/transduction

All cell culture media were from Invitrogen (Breda, The Netherlands). MEF cells devoid of Gαq/11 subunits [33], derived from Gαq/11 knockout mice were cultured in Dulbecco's modified Eagle's medium (DMEM) (#21969-035)] supplied with 1× non-essential amino acids, 10% fetal bovine serum (FBS), 1× L-glutamine, 100 U/ml penicillin and 100 μg/ml streptomycin. For transduction of these cells, the retroviral vector pBABE-Gαq-YFP was transfected into Phoenix-Eco package cells, and the supernatant, containing viral particles, was harvested 48 hours after infection. Cells were incubated with 1 ml of this supernatant in the presence of 10 μl Dotap (1 mg/ml) (Roche, Indianapolis, IN, USA) and after 48 hours, cells were grown for 2 weeks on media containing 2 μg/ml puromycin for selection. For imaging, these cells were cultured on glass coverslips and loaded with the calcium indicator Fura Red (6 μg/ml final concentration) (Fura Red AM; Molecular Probes, Eugene, OR, USA) for 30 minutes at room temperature. For imaging, non-transduced MEF cells were cultured on glass coverslips and transfected (Lipofectamine reagent; Invitrogen) with plasmids encoding Gαq-mTqΔ6. After overnight incubation at 37°C and 5% CO_2_, the cells were loaded with Fura Red and mounted in a cell chamber (Attofluor; Invitrogen), submerged in medium (140 mmol/l NaCl, 5 mmol/l KCl, 1 mmol/l MgCl_2_, 1 mmol/l CaCl_2_, 10 mmol/l glucose, 20 mmol/l HEPES pH 7.4) and viewed under a confocal microscope.

HeLa cells (American Tissue Culture Collection; Manassas, VA, USA) were cultured in DMEM plus Glutamax (#61965-059), 10% FBS, 100 U/ml penicillin and 100 μg/ml streptomycin. Transfection and preparation for imaging purposes were similar to the procedures described above for MEF cells.

### Confocal microscopy

Mammalian cells were imaged using a confocal laser-scanning microscope (LSM 510; Carl Zeiss GmbH, Oberkochen, Germany) with an oil-immersion objective (Plan-A 63×/1.4; Carl Zeiss GmbH). Samples were excited with a 488-nm argon laser. For YFP/Fura Red, the following settings were used a primary dichroic mirror (488 nm) and a secondary dichroic mirror (570 nm), thereby splitting the Fura Red fluorescence from the YFP fluorescence. The 505 to 550 nm bandpass filter was used to yield the YFP image, and the long-pass 650 nm filter was used to obtain the Fura Red image. MEF cells were stimulated with 1 μmol/l (final concentration) BK (Sigma-Aldrich, St. Louis, MO, USA), and the Fura Red intensity was followed in time as a measure of intracellular calcium levels. The n determinations are derived from different cells, expressing different amounts of protein from the same batch of MEF cells on different days, and throughout the study, cells from different experiments were pooled and are displayed as n determinations.

Fast imaging was performed on a confocal microscope (A1; Nikon, Tokyo, Japan). The excitation light was from a 443-nm laser, which was reflected onto the sample by a 457/514 dichroic mirror. Fluorescence emission was separated by a secondary dichroic mirror (515 nm). mTq fluorescence was filtered through a 464 to 499 nm emission filter, and sensitized emission was filtered through a 525 to 555 nm emission filter. A 40× objective was used, and the pinhole was completely opened. The frame size was 512 × 128 pixels, and images were acquired at 15 frames per second (60 frames/s with four line averages). The curve was fitted using

The half-time of the first-order reaction is given by:

### Wide-field fluorescence microscopy

Ratiometric FRET measurements in HeLa cells were performed using a wide-field fluorescence microscope (Axiovert 200 M; Carl Zeiss GmbH) kept at 37°C, equipped with an oil-immersion objective (Plan-Neofluar 40×/1.30; Carl Zeiss GmbH) and a xenon arc lamp with monochromator (Cairn Research, Faversham, Kent, UK). Images were recorded with a cooled charged-coupled device camera (Coolsnap HQ, Roper Scientific, Tucson, AZ, USA). Fluorophores were excited with 420 nm light (slit width 30 nm), mTq emission was detected with the bandpass 470/30 filter, and YFP emission was detected with the BP535/30 filter by turning the filter wheel. The exposure time for each image was 200 ms. HeLa cells were stimulated with 100 μmol/l (final concentration) histamine (Sigma-Aldrich) and mTq/YFP emission was followed in time. Pyrilamine (mepyramine) (Sigma-Aldrich) was added to obtain a final concentration of 10 μmol/l.

### Fluorescence lifetime imaging microscopy

Frequency-domain FLIM measurements were performed using the apparatus described previously [66], equipped with an oil-immersion objective (Plan Apochromat 63×/1.4 objective; Carl Zeiss GmbH). Samples were excited by means of a 442 nm helium-cadmium laser modulated at 75.1 MHz and a BP 480/40 emission filter was used to detect mTq fluorescence. FLIM stacks of 24 phase images permutated in recording order [67] were acquired with an exposure time of about 0.1 to 0.5 seconds each. FRET efficiency was calculated as follows:

where τ_DA _and τ_D _are the donor lifetimes in the presence and absence of an acceptor, respectively.

### Membrane stretch

A hypotonic stimulus was applied by replacing 0.5 ml medium by 20 mmol/l HEPES pH 7.4.

### WB

The antibody against Gαq/11 (sc-46972, C-16; Santa Cruz Biochemicals, Santa Cruz, CA, USA) was directed against an epitope near the C terminus.

### H1R expression level determination

HeLa cells were scraped from a 90 mm petri dish, and resuspended in binding buffer (50 mmol/l Na_2_/K-phosphate buffer pH 7.4), and mixed by sonication to ensure a homogenous membrane suspension. The membrane suspension was added to premixed radioligand/competitor or radioligand/buffer solutions, and incubated for approximately 1 hour at room temperature on a shaking table (750 rpm). Free radioligand was separated from bound radioligand by filtration through a GF/C filterplate (PerkinElmer Corp., Waltham, MA, USA) pre-soaked with 0.5% polyethyleneimine. Scintillation fluid was added to the filter plate, and radioactivity was measured in a Wallac micro β counter. Each well contained 25 μl competitor (final concentration: 10 μmol/l mianserin) or binding buffer, 25 μl radioligand (25.8 Ci/mmol [^3^H]-mepyramine) and 50 μl membrane suspension.

## Authors' contributions

MAH designed the experiments, acquired and analyzed the data, and wrote the manuscript. JG designed the experiments, acquired the data, and was involved in the writing/revision of the manuscript. LvW constructed Gα_q_-mTqΔ6 and performed western blot analyses. SN determined the H1R expression levels. EMM provided technical assistance with the microscope (Nikon A1). SO provided the MEFq/11^-/- ^cell line, gave advice with respect to experiments, and was involved in the revision of the manuscript.,TWJG designed the experiments, and was involved in the writing and revision of the manuscript. All authors read and approved the final manuscript.

## Supplementary Material

Additional File 1 **Gαq-monomeric visible fluorescent protein (mVFP) expression in various cell lines (A) **Madin-Darby canine kidney (MDCK) cells expressing Gαq-monomeric yellow fluorescent protein (mYFP); **(B) **N1E-115 neuroblastoma cells expressing Gαq-mYFP; **(C) **HEK293 cells expressing Gαq-mYFP; **(D) **HeLa cells expressing Gαq-mTqΔ6. Click here for file

Additional File 2 **Expression of Gαq in mouse embryonic fibroblasts (MEF) and HeLa cell lines **Transfected, transduced or untreated cells were treated with trypsin and lysed. Each lane was loaded with 40 μg of protein. Western blotting was performed with an antibody against Gαq/11 (diluted 1:1000) and exposed for 30 seconds. Lane 1 contains wild-type HeLa cells; lane 2 contains HeLa cells transfected with Gαq-mTqΔ6; lane 3 contains MEFq/11^-/- ^cells; lane 4 contains MEFq/11^-/- ^cells transduced with Gαq-mYFP and lane 5 contains wild-type MEF cells. Click here for file

Additional File 3 **Expression of Gαq-monomeric Turquoise (mTq)Δ6 versus Gαq-enhanced cyan fluorescent protein (ECFP) in HeLa cells **Representative images of HeLa cells expressing Gαq-ECFP (top) or Gαq-mTqΔ6 (bottom), 2 days after transfection with equal amounts of DNA. The images were acquired with the same exposure time and contrast settings. Click here for file

Additional File 4 **Gq fluorescence resonance energy transfer (FRET) movie **Movie displaying the change in FRET ratio upon addition of histamine (100 μmol/l), followed by the addition of mepyramine (10 μmol/l) in HeLa cells expressing Gαq-monomeric Turquoise (mTq)Δ6, Gβ1, yellow fluorescent protein (YFP)-Gγ2 and histamine 1 receptor (H1R). Click here for file

Additional File 5 **Long-term activation of Gq **Graph depicting the duration of the change in fluorescence resonance energy transfer (FRET) ratio (yellow fluorescent protein:monomeric Turquoise (YFP:mTq)) in the continuous presence of histamine in HeLa cells expressing Gαq-monomeric Turquoise (mTq)Δ6, Gβ1, YFP-Gγ2 and histamine 1 receptor (H1R). Click here for file

Additional File 6 **Regulators of G-protein signalling (RGS)-sensitive versus RGS-insensitive Gq (A) **The fluorescence resonance energy transfer (FRET) ratio change (yellow fluorescent protein:monomeric Turquoise (YFP:mTq)) upon addition of histamine (100 μmol/l) in HeLa cells expressing Gαq-mTqΔ6, Gβ1 and YFP-Gγ2 (n = 4; error bars depict SE). **(B) **The FRET ratio change (YFP:mTq) upon addition of histamine (100 μmol/l) in HeLa cells expressing Gαq-mTqΔ6-G188S, Gβ1 and YFP-Gγ2 (n = 8). Click here for file

Additional File 7 **Effect of mepyramine on Gq fluorescence resonance energy transfer (FRET) ratio changes in the presence of the guanine nucleotide exchange factor p63RhoGEF **Representative trace depicting the FRET ratio change (yellow fluorescent protein:monomeric Turquoise (YFP:mTq)) upon addition of histamine (100 μmol/l) in HeLa cells coexpressing Gαq-mTqΔ6, Gβ1, YFP-Gγ2 and p63RhoGEF. Addition of the histamine 1 receptor (H1R) inverse agonist mepyramine (10 μmol/l) reversed the ratio change induced by histamine. (Inset) the mTq and YFP intensity traces from which the ratio was derived. Click here for file

Additional File 8 **Overview of observed fluorescence resonance energy transfer (FRET) ratio changes **Histogram depicting the normalized FRET ratio (yellow fluorescent protein:monomeric Turquoise (YFP:mTq)) changes upon histamine stimulation, observed in cells expressing Gαq-mTqΔ6, Gβ1 and YFP-Gγ2 (wild-type (wt); n = 54); cells expressing Gαq-mTqΔ6, Gβ1, YFP-Gγ2 and histamine 1 receptor (H1R, n = 8); cells expressing Gαq-mTqΔ6, Gβ1, YFP-Gγ2 and the guanine nucleotide exchange factor p63RhoGEF (p63, n = 29); cells expressing Gαq-mTqΔ6, Gβ1, YFP-Gγ2 and p63RhoGEF-L475A (p63LA, n = 15). The error bars depict SE. Click here for file

Additional File 9 **Lifetime data (A) **Gαq-monomeric Turquoise (mTq)Δ6 intensity **(C) **and phase lifetime image in the absence of acceptor (yellow fluorescent protein) YFP-Gγ2. **(B) **Gαq-mTqΔ6 intensity and **(D) **phase lifetime image in the presence of acceptor YFP-Gγ2. **(E) **Phase lifetime histogram; lifetime distribution of the depicted cells in the presence (left population) and in the absence of the acceptor (right population). **(F-J) **Gαq-mTqΔ6 intensity image in the presence of acceptor YFP-Gγ_2 _and histamine 1 receptor (H1R) **(F) **before and **(G) **after addition of histamine. Phase lifetime image of Gαq-mTqΔ6 **(H) **before and **(I) **after addition of histamine. **(J) **Phase lifetime histogram; lifetime distribution of the depicted cells (left) before and (right) after addition of histamine. **(K-O) **Gαq-mTqΔ6 intensity image in the presence of acceptor YFP-Gγ_2 _and the guanine nucleotide exchange factor p63-RhoGEF **(K) **before and **(L) **after addition of histamine. Phase lifetime image of Gαq-mTqΔ6 **(M) **before and **(N) **after addition of histamine. **(O) **Phase lifetime histogram; lifetime distribution of the depicted cells (left) before and (right) after addition of histamine. Click here for file

Additional File 10 **PtdIns(4,5)P**_**2 **_**hydrolysis upon hypotonic stimulus (A) **Green fluorescent protein (GFP)-PH was used as an indicator for PtdIns(4,5)P2 levels, and was found to be localized predominantly at the plasma membrane of HeLa cells. **(C) **Membrane localization was quantified by means of a line profile plot. Upon applying the hypotonic stimulus **(B) **the PH domain translocated partially to the cytoplasm, which is also evident from **(D) **the line profile plot, showing decreased membrane intensity and increased cytoplasmic intensity. Click here for file
