# Supplementary figures and images for: Real-time visualization of heterotrimeric G protein Gq activation in living cells
Source: BMC Biol. 2011 May 27;9:32. doi: 10.1186/1741-7007-9-32 (PMC3129320; doi:10.1186/1741-7007-9-32)

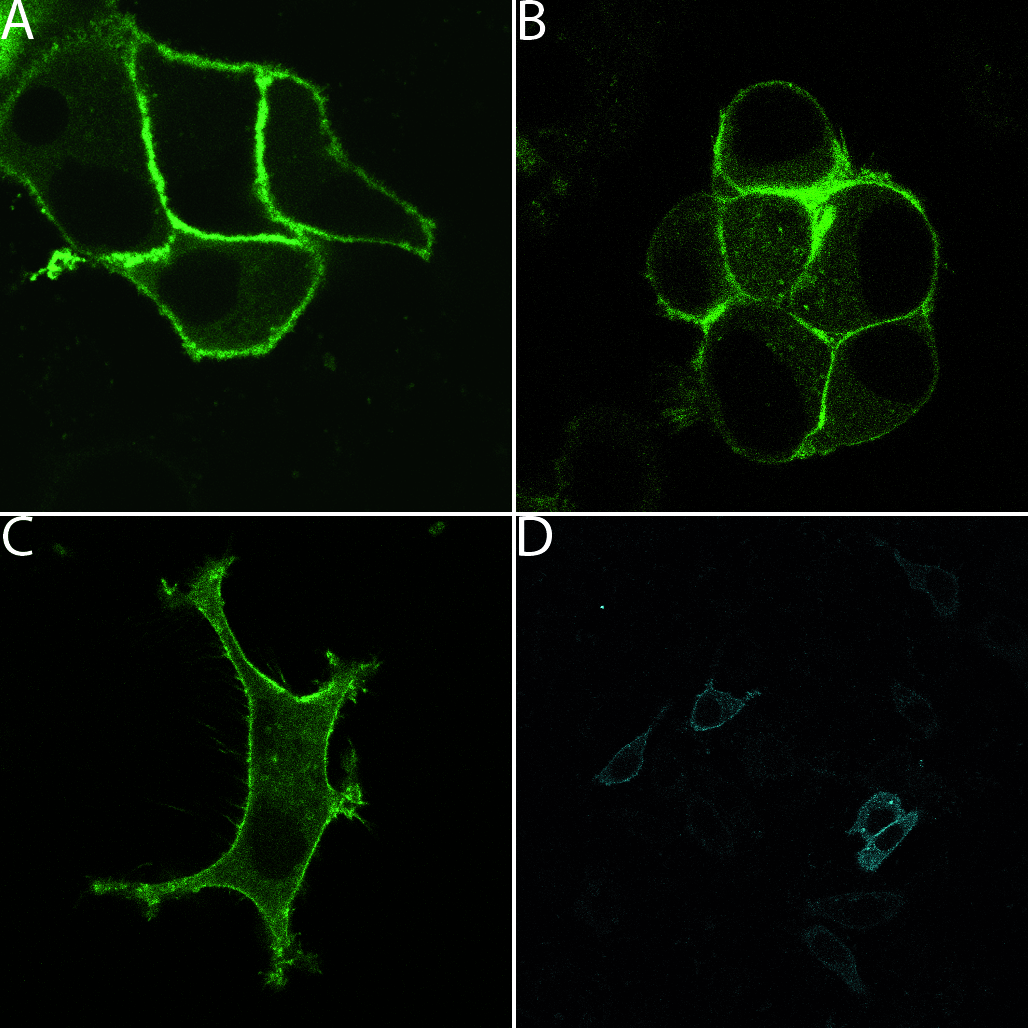

Supplement: Additional File 1 — Gαq-monomeric visible fluorescent protein (mVFP) expression in various cell lines (A) Madin-Darby canine kidney (MDCK) cells expressing Gαq-monomeric yellow fluorescent protein (mYFP); (B) N1E-115 neuroblastoma cells expressing Gαq-mYFP; (C) HEK293 cells expressing Gαq-mYFP; (D) HeLa cells expressing Gαq-mTqΔ6. [file 1741-7007-9-32-S1.TIFF]

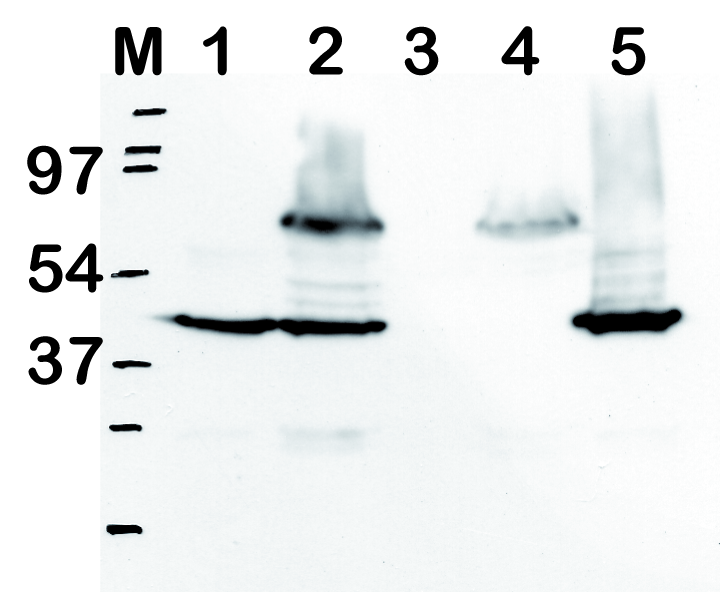

Supplement: Additional File 2 — Expression of Gαq in mouse embryonic fibroblasts (MEF) and HeLa cell lines Transfected, transduced or untreated cells were treated with trypsin and lysed. Each lane was loaded with 40 μg of protein. Western blotting was performed with an antibody against Gαq/11 (diluted 1:1000) and exposed for 30 seconds. Lane 1 contains wild-type HeLa cells; lane 2 contains HeLa cells transfected with Gαq-mTqΔ6; lane 3 contains MEFq/11-/- cells; lane 4 contains MEFq/11-/- cells transduced with Gαq-mYFP and lane 5 contains wild-type MEF cells. [file 1741-7007-9-32-S2.TIFF]

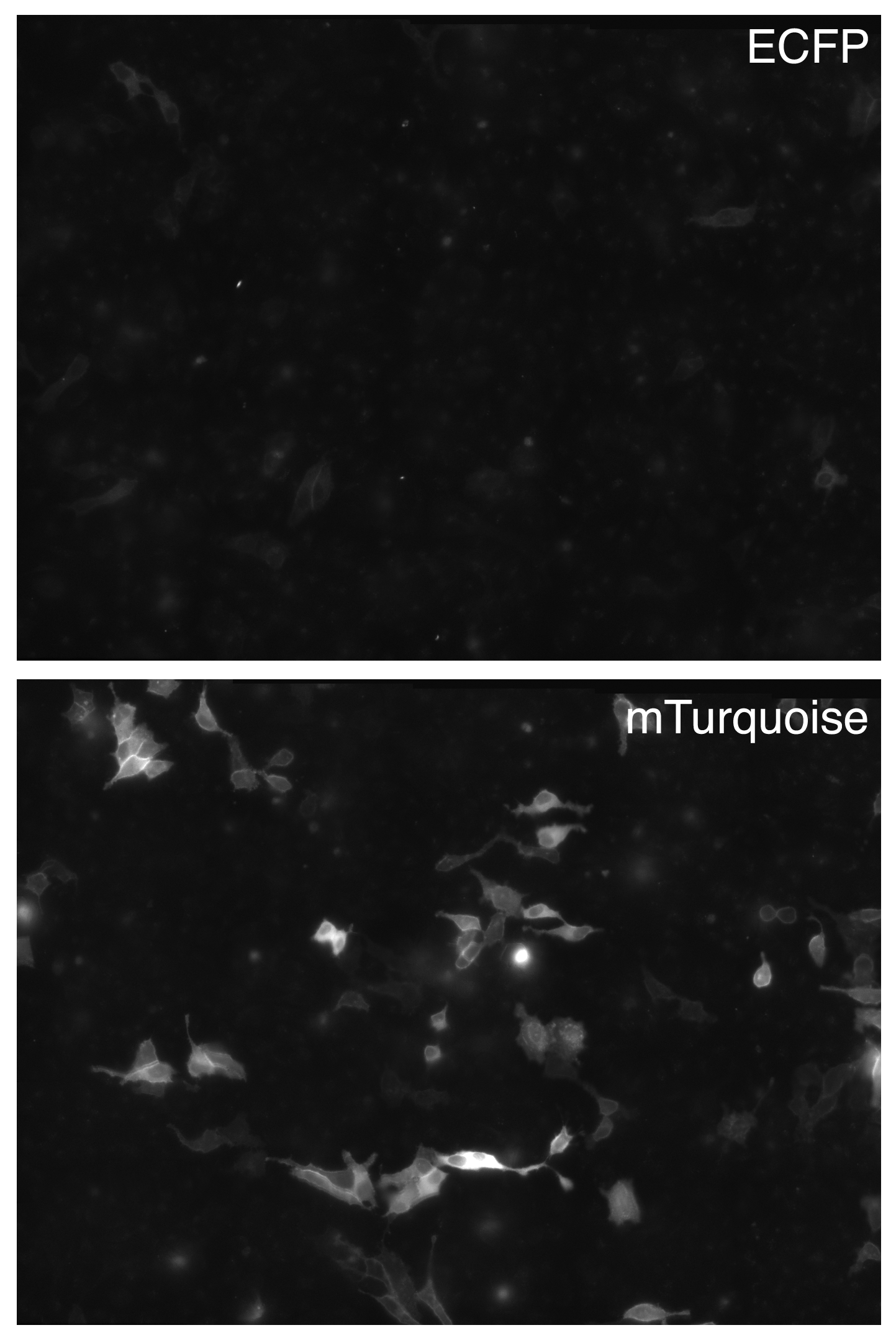

Supplement: Additional File 3 — Expression of Gαq-monomeric Turquoise (mTq)Δ6 versus Gαq-enhanced cyan fluorescent protein (ECFP) in HeLa cells Representative images of HeLa cells expressing Gαq-ECFP (top) or Gαq-mTqΔ6 (bottom), 2 days after transfection with equal amounts of DNA. The images were acquired with the same exposure time and contrast settings. [file 1741-7007-9-32-S3.TIFF]

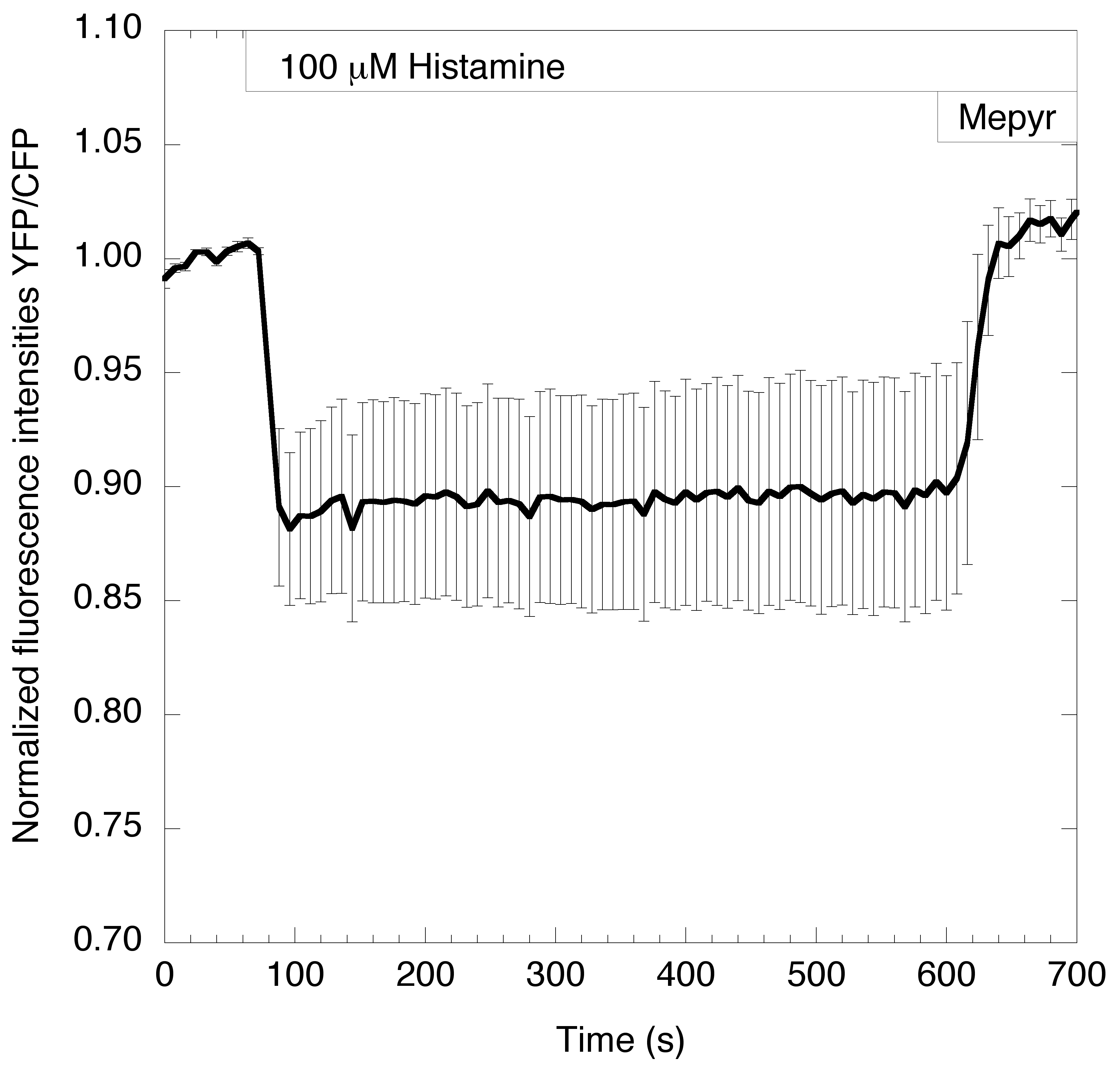

Supplement: Additional File 5 — Long-term activation of Gq Graph depicting the duration of the change in fluorescence resonance energy transfer (FRET) ratio (yellow fluorescent protein:monomeric Turquoise (YFP:mTq)) in the continuous presence of histamine in HeLa cells expressing Gαq-monomeric Turquoise (mTq)Δ6, Gβ1, YFP-Gγ2 and histamine 1 receptor (H1R). [file 1741-7007-9-32-S5.TIFF]

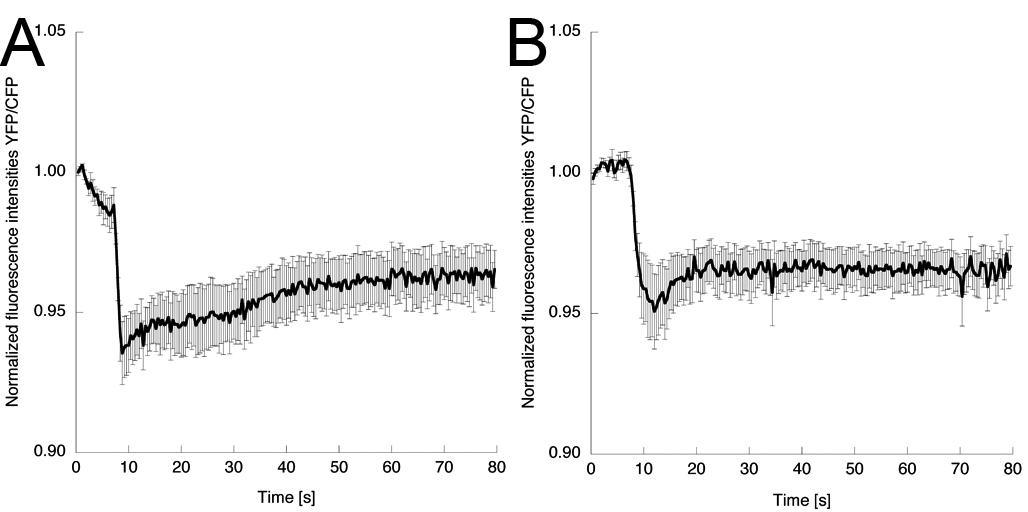

Supplement: Additional File 6 — Regulators of G-protein signalling (RGS)-sensitive versus RGS-insensitive Gq (A) The fluorescence resonance energy transfer (FRET) ratio change (yellow fluorescent protein:monomeric Turquoise (YFP:mTq)) upon addition of histamine (100 μmol/l) in HeLa cells expressing Gαq-mTqΔ6, Gβ1 and YFP-Gγ2 (n = 4; error bars depict SE). (B) The FRET ratio change (YFP:mTq) upon addition of histamine (100 μmol/l) in HeLa cells expressing Gαq-mTqΔ6-G188S, Gβ1 and YFP-Gγ2 (n = 8). [file 1741-7007-9-32-S6.TIFF]

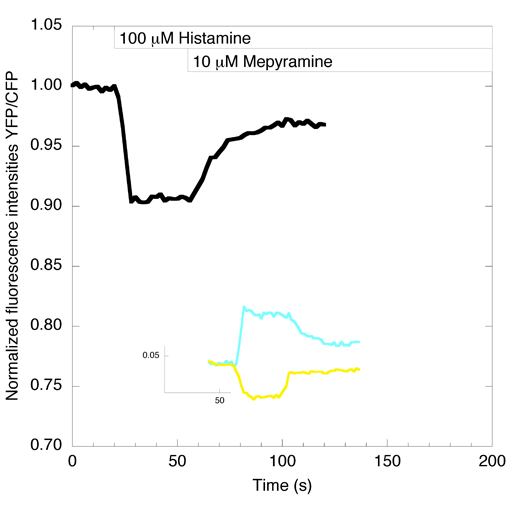

Supplement: Additional File 7 — Effect of mepyramine on Gq fluorescence resonance energy transfer (FRET) ratio changes in the presence of the guanine nucleotide exchange factor p63RhoGEF Representative trace depicting the FRET ratio change (yellow fluorescent protein:monomeric Turquoise (YFP:mTq)) upon addition of histamine (100 μmol/l) in HeLa cells coexpressing Gαq-mTqΔ6, Gβ1, YFP-Gγ2 and p63RhoGEF. Addition of the histamine 1 receptor (H1R) inverse agonist mepyramine (10 μmol/l) reversed the ratio change induced by histamine. (Inset) the mTq and YFP intensity traces from which the ratio was derived. [file 1741-7007-9-32-S7.TIFF]

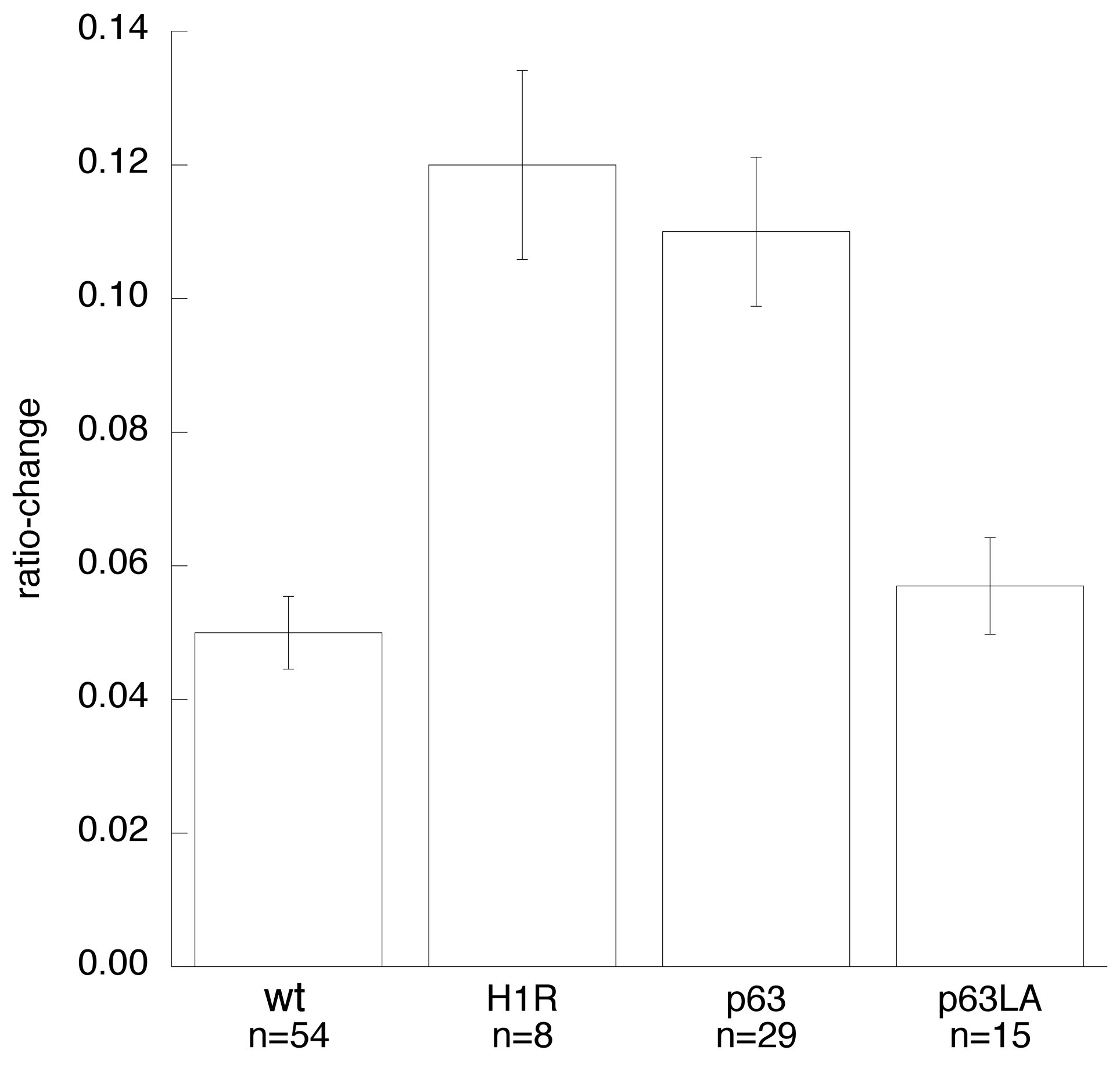

Supplement: Additional File 8 — Overview of observed fluorescence resonance energy transfer (FRET) ratio changes Histogram depicting the normalized FRET ratio (yellow fluorescent protein:monomeric Turquoise (YFP:mTq)) changes upon histamine stimulation, observed in cells expressing Gαq-mTqΔ6, Gβ1 and YFP-Gγ2 (wild-type (wt); n = 54); cells expressing Gαq-mTqΔ6, Gβ1, YFP-Gγ2 and histamine 1 receptor (H1R, n = 8); cells expressing Gαq-mTqΔ6, Gβ1, YFP-Gγ2 and the guanine nucleotide exchange factor p63RhoGEF (p63, n = 29); cells expressing Gαq-mTqΔ6, Gβ1, YFP-Gγ2 and p63RhoGEF-L475A (p63LA, n = 15). The error bars depict SE. [file 1741-7007-9-32-S8.TIFF]

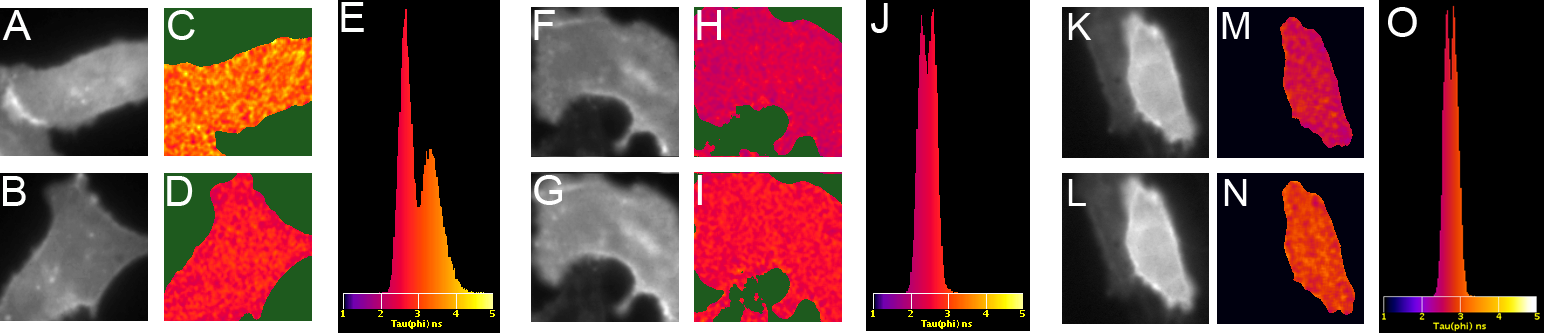

Supplement: Additional File 9 — Lifetime data (A) Gαq-monomeric Turquoise (mTq)Δ6 intensity (C) and phase lifetime image in the absence of acceptor (yellow fluorescent protein) YFP-Gγ2. (B) Gαq-mTqΔ6 intensity and (D) phase lifetime image in the presence of acceptor YFP-Gγ2. (E) Phase lifetime histogram; lifetime distribution of the depicted cells in the presence (left population) and in the absence of the acceptor (right population). (F-J) Gαq-mTqΔ6 intensity image in the presence of acceptor YFP-Gγ2 and histamine 1 receptor (H1R) (F) before and (G) after addition of histamine. Phase lifetime image of Gαq-mTqΔ6 (H) before and (I) after addition of histamine. (J) Phase lifetime histogram; lifetime distribution of the depicted cells (left) before and (right) after addition of histamine. (K-O) Gαq-mTqΔ6 intensity image in the presence of acceptor YFP-Gγ2 and the guanine nucleotide exchange factor p63-RhoGEF (K) before and (L) after addition of histamine. Phase lifetime image of Gαq-mTqΔ6 (M) before and (N) after addition of histamine. (O) Phase lifetime histogram; lifetime distribution of the depicted cells (left) before and (right) after addition of histamine. [file 1741-7007-9-32-S9.TIFF]

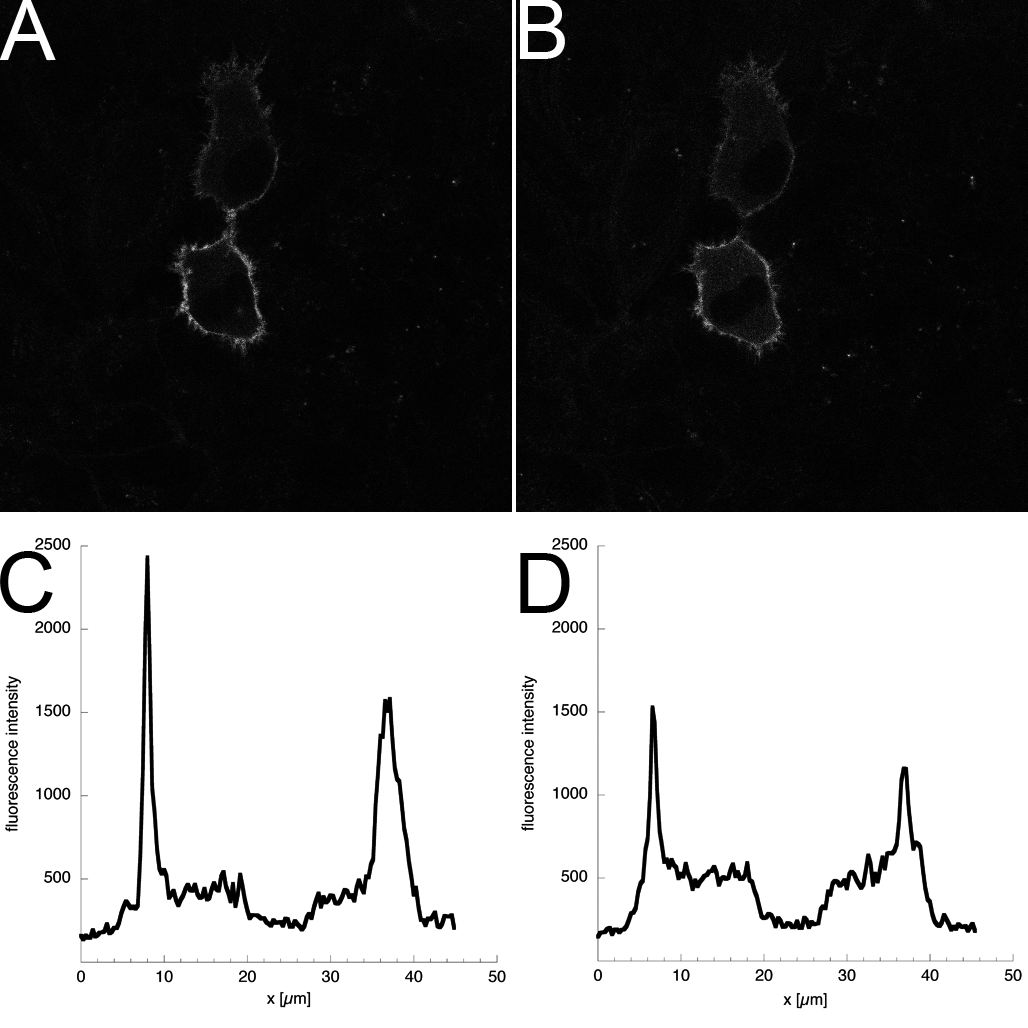

Supplement: Additional File 10 — PtdIns(4,5)P2 hydrolysis upon hypotonic stimulus (A) Green fluorescent protein (GFP)-PH was used as an indicator for PtdIns(4,5)P2 levels, and was found to be localized predominantly at the plasma membrane of HeLa cells. (C) Membrane localization was quantified by means of a line profile plot. Upon applying the hypotonic stimulus (B) the PH domain translocated partially to the cytoplasm, which is also evident from (D) the line profile plot, showing decreased membrane intensity and increased cytoplasmic intensity. [file 1741-7007-9-32-S10.TIFF]
